# Bridging the Divide in India’s Tobacco Epidemic: A Review of the Effectiveness of Four Core Policy Interventions

**DOI:** 10.1155/ijod/1393877

**Published:** 2026-10-06

**Authors:** Rekha P. Shenoy, T. A. Abdul Salam, M. Alfiya, Amanna Supriya

**Affiliations:** ^1^ Department of Public Health Dentistry Yenepoya Dental College Yenepoya (Deemed to be University) University Road, Deralakatte Mangalore 575018 Karnataka India yenepoya.edu.in; ^2^ Department of Public Health Dentistry PSM College of Dental Science and Research Thrissur 680519 Kerala India

**Keywords:** effectiveness, policies, tobacco control

## Abstract

**Introduction:**

Tobacco is one of the major risk factors for non‐communicable diseases (NCDs) in the Indian population. The decreasing trend of tobacco use reflects significant progress in tobacco control, which can be attributed to key policies like price rise, smoke‐free areas, ban on advertising and promotion and pictorial warnings on tobacco products. This narrative review was undertaken to assess effectiveness of these four policies in reducing tobacco burden in India.

**Materials and Methods:**

Articles were obtained by searching through all major databases. Peer‐reviewed articles published in the last 20 years, reports of the Government of India and nationally representative surveys focusing on at least one intervention were included. Selected publications and reports went through two‐stage screening process before inclusion. Findings were grouped according to the four policies under evaluation.

**Main Text:**

Twenty‐three articles and reports were selected to derive the results. Inverse association was found between product price and consumption/affordability. Smoke‐free laws in public spaces were associated with reduced second‐hand smoke (SHS) exposure at home. Point of sale (POS) advertising remains prominent in India, which is a cause for concern. Awareness about pictorial warnings was significantly high among both consumers and non‐consumers who believed they heightened awareness about health hazards and assisted in reducing or quitting use. However, high‐dependence users were not influenced by statutory warnings or price increases.

**Conclusions:**

To achieve full compliance with COTPA (The Cigarettes and Other Tobacco Products [Prohibition of Advertisement and Regulation of Trade and Commerce, Production, Supply and Distribution] Act), 2003, multisectoral interventions will require partnership of government agencies, civil society organisations and the community. While tobacco products are taxed heavily to make them less affordable and smoke‐free legislation have reduced SHS exposure, advertising in all forms should be prohibited and pictorial warnings mandated on cigarette sticks. Implementing tobacco endgame strategies may provide the critical breakthrough.

## 1. Introduction

Tobacco is one of the leading causes of death and disability in the Indian population, besides being one of the major risk factors implicated in non‐communicable disease (NCD) aetiology. The three main diseases caused by tobacco use are cancer, coronary artery disease and chronic obstructive lung disease. The Global Burden of Disease (GBD) Study reported over 1.2 million deaths due to smoking and second‐hand smoke (SHS) in India in 2019. The World Health Organisation (WHO) attributes an estimated 1.35 million deaths in India every year to tobacco use [1].

Data obtained from several national surveys conducted across India show a decreasing trend in tobacco use. According to the Global Adult Tobacco Survey (GATS), current overall tobacco use decreased from 34.6% (GATS‐1, 2009–2010) to 28.6% (GATS‐2, 2016–2017), and tobacco use among minors (15–17 years) decreased from 10% (GATS‐1, 2009–2010) to 4% (GATS‐2, 2016–2017). The Global Youth Tobacco Survey (GYTS) also found that current tobacco use among 13–15‐year‐olds declined from 14.6% (GYTS 2010) to 8.4% (GYTS 2019). The National Family Health Survey (NFHS‐5, 2019–2021) reported 15.1% and 4.4% of men and women aged 15 years and above, respectively, using any kind of tobacco [1–4].

In 2020, the total land under tobacco cultivation in India was 4.5 lakh hectares (a meagre 0.26% of total arable land), and the total production was 7.6 lakh tons. The average annual revenue from tobacco products from 2018 to 2021 was Rs. 53,750 crores. Exports of tobacco stood at Rs. 6510 crores in 2020–2021. However, the estimated cost of diseases and deaths attributed to tobacco use in India in 2017–2018 among those aged 35 years and above stood at Rs. 177,341 crores, which was more than 1% of the gross domestic product (GDP) that year, while excise revenues from tobacco in 2016–2017 were just 12.2% of this cost [1].

India has shown significant progress in tobacco control since 2004, when the COTPA (The Cigarettes and Other Tobacco Products [Prohibition of Advertisement and Regulation of Trade and Commerce, Production, Supply and Distribution] Act), 2003, came into force. In 2004, India ratified the WHO’s Framework Convention on Tobacco Control (FCTC), and the National Tobacco Control Programme (NTCP) was introduced in 2007–2008. In April 2016, pictorial health warnings (PHWs) were enhanced from 40% to 85% of the pack surface. In September 2018, tobacco packs in India displayed new rotated 85% PHWs with the National Tobacco Control Quitline number. In 2019, the Prohibition of Electronic Cigarettes (Production, Manufacture, Import, Export, Transport, Sale, Distribution, Storage and Advertisement) Act was passed. On the taxation front, the Government of India (GoI) in 2017 levied 5% Goods and Services Tax (GST) on raw tobacco and 28% GST on all tobacco products. An additional compensation cess was levied on cigarettes and smokeless tobacco (SLT) products. However, GST provisions exempt industries with an annual turnover below Rs. 40 lakh and a significant part of the bidi industry (being under the informal sector) continues to remain outside the tax net [1].

While tobacco use has decreased, India continues to be the world’s second largest consumer and producer of tobacco, accounting for 13% of the total global production in 2020 [1, 5]. Tobacco production in India is significantly more than its domestic consumption. While ~267 million adults consume tobacco, the country produces a surplus that makes it the second‐largest exporter of unmanufactured tobacco globally. Tobacco use is unacceptably high among adolescents and adults, with India having the largest number of SLT users in the world. For every Rs. 100 received as excise tax from tobacco products, Rs. 816 of costs are imposed on society through its consumption. The tobacco industry overplays its contribution to the Indian economy, with mass media campaigns featuring images of poor farmers and inflated estimates of its contributions, ignoring health costs and exploitative labour practices [1]. Therefore, this narrative review was undertaken to assess the effectiveness of four key tobacco control policies in reducing the tobacco burden in India.

## 2. Materials and Methods

This narrative review aimed to assess the effectiveness of four tobacco‐control policies in India—price rise, smoke‐free areas, ban on advertising and promotion and pictorial warnings on tobacco products. A comprehensive literature search was conducted to identify relevant studies published over the past two decades.

### 2.1. Literature Search Strategy

Multiple electronic databases were systematically searched, including PubMed, Scopus, Web of Science, Embase and Google Scholar. In addition, ProQuest Central and Clinical Key Flex were also accessed. The search covered studies published from January 2006 to December 2025 to capture contemporary evidence on tobacco‐control policies in India. Additional sources, such as reference lists of included publications and relevant government reports, were also manually screened to ensure completeness.

A combination of keywords and Boolean operators was used to develop the search strategy. Key terms included ‘tobacco control’, ‘India’, ‘tobacco price rise’, ‘taxation’, ‘smoke‐free laws’, ‘smoke‐free areas’, ‘advertising ban’, ‘tobacco marketing restrictions’, ‘pictorial health warnings’ and ‘policy effectiveness’. These terms were combined using ‘AND’ and ‘OR’ operators and adapted as needed for each database.

The publications thus obtained were screened in two stages. First, all relevant publications were retrieved from the selected databases, and title and abstracts were screened to identify potentially relevant studies. In the second stage, the full texts of the shortlisted publications were assessed in detail against the predefined inclusion and exclusion criteria. Of the 53 identified publications, 36 were deemed relevant at the end of stage one, out of which 22 fulfilled the selection criteria and formed the basis of the review.

Publications were included if they:

• Focused on tobacco‐control policies implemented in India.
• Evaluated at least one of the following interventions: price rise/taxation, smoke‐free policies, advertising bans or PHWs.
• Reported outcomes related to policy effectiveness, such as changes in tobacco use prevalence, awareness, policy compliance or enforcement, quit or initiation rates.
• Were reports of the GoI, and national representative surveys conducted by the GoI with tobacco as a component.
• Were published in peer‐reviewed journals available in English between January 2006 and December 2025.
• Included policy analyses, programme evaluations and reviews.
• Were available in full text.

Publications were excluded if they:

• Addressed tobacco use without evaluating policy interventions.
• Lacked clear methodology or outcome measures related to policy effectiveness.
• Were duplicate publications, retracted publications or inaccessible in full text.

### 2.2. Data Extraction and Synthesis

Relevant data from included publications were extracted, including study design, population characteristics, type of tobacco‐control policy evaluated, outcome measures, and key findings. Findings were grouped thematically according to the four policy domains (price rise, smoke‐free areas, ban on advertising and promotion and pictorial warnings on tobacco products), allowing for a structured comparison of evidence across studies. This approach enabled a contextualised understanding of the effectiveness of these four tobacco‐control policies in India over the past two decades.

## 3. Main Text

Twenty‐two publications and reports fulfilled the criteria and were analyzed to derive the results of this narrative review. They were evaluated on the four policies under review. India’s tobacco control law prohibits smoking in public places and indoor workplaces (Section 4); tobacco advertising, promotion, and sponsorships except at the point of sale (POS), on‐pack and in‐pack (Section 5); and makes it obligatory to display PHWs (Section 7) [1, 6–8].

### 3.1. Price Rise (Covered Under FCTC Article 6)

It is not covered under the COTPA as it is a health regulation law, not a fiscal or price control policy. Pricing and taxation of tobacco products are governed by the Ministry of Finance, and tobacco products have been included in the ‘sin goods’ category with the highest tax slab in alignment with the WHO FCTC to make them less affordable [1, 8]. The perceived effect of increased pricing on smoked tobacco product quit rates in Haryana found that 74% current smokers stated intent to quit smoking if price was increased, with higher odds of quitting among cigarette than bidi smokers (OR = 5.2) [9]. However, smoked and SLT users with high dependence in Kanpur reported that they were not influenced by the price hike [10].

A systematic review on the impact of tobacco price and taxation on affordability and consumption of tobacco products in the South‐East Asian Region, in which 11 of the 28 studies reviewed were from India, reported an inverse association between price and consumption/affordability, with the less affluent more price responsive compared to the more affluent [11]. The price rise through increased taxation is one of the most effective measures in tobacco control, directly influencing behaviour, health outcomes and social equity. Higher taxes reduce affordability by increasing the price of the product. This is grounded in the principle of price elasticity of demand (as prices rise, consumption falls). Hence, taxation is particularly powerful in preventing initiation and encouraging quitting by acting as a barrier to experimentation and addiction. It also encourages cessation among current users. Taxation reduces the health and economic burden by reducing healthcare costs, loss of productivity and premature deaths, especially among the low‐income groups. Also, differential taxation across tobacco products may result in users switching to cheaper alternatives like bidis instead of quitting. This is a major policy challenge for India. Bidis are widely consumed but lightly taxed as they are produced in the informal sector, which is difficult to regulate. The availability of a wide range of products in India aids users in switching between cigarettes, bidis and SLT. Policy changes advocating uniform taxation across all tobacco products are critical to stop product substitution. In India, tobacco taxation works through a high GST rate plus a compensation cess, making it one of the central tools for tobacco control. While effective for cigarettes, uneven taxation across products limits their full impact. Strengthening and harmonising tax policies remain the key to better public health outcomes.

Ideally, tax and price increases should outpace inflation and income growth to remain effective. Rising incomes can make tobacco remain affordable despite high taxation [11]. An analysis of the affordability of cigarettes and bidis in India at different taxation rates (0%–200%) found that bidis were more affordable at lower increments in tax when compared to cigarettes. For cigarettes, a fall in affordability was seen at a 100% tax increase, whereas for bidis, a fall in affordability was only seen at a 150%–200% increase in tax [12]. Therefore, an appropriate increase in the taxation of bidis can make them less affordable.

### 3.2. Smoke‐Free Areas (Covered Under FCTC Article 8, COTPA Section 4)

Data from GATS‐1, 2009–2010, was analysed to check the association between working in a smoke‐free indoor environment and living in a smoke‐free home. Indian states with higher proportions of smoke‐free workplaces had higher proportions of smoke‐free homes [13]. Comparison of smoking at home in two jurisdictions in India—a compliance‐validated smoke‐free jurisdiction (Shimla in 2010) versus a non‐smoke‐free jurisdiction (Haridwar) between 2009 and 2015—revealed that smoke‐free laws in public spaces were associated with reduced SHS exposure at home [14].

Exposure to SHS inside homes ranged from 3% to 46%, while SHS exposure in enclosed public places ranged from 8% to 65% across all states and union territories in India [3, 4]. There was a significant increase in the number of smoke‐free homes (44% in GATS‐2 versus 35% in GATS‐1) with South India having the highest proportion of smoke‐free homes, which were associated with higher education levels, higher wealth quintiles and urban residence. While 41% of respondents reported smoke‐free workplaces, 26.2% indoor workers and 30.2% adults reported SHS exposure at their workplace [2].

Smoke‐free homes and public places are a direct pointer towards successful implementation of tobacco control laws. It is heartening to note the definitive increase in the prevalence of smoke‐free homes over the past decades. Smoke‐free environments are vital for public health, protecting non‐smokers toxic effects of second‐hand and third‐hand smoke and their after‐effects. They improve air quality, encourage smokers to quit, prevent initiation, and offer economic benefits by lowering healthcare costs.

### 3.3. Ban on Advertising and Promotion (Covered Under FCTC Article 13, COTPA Section 5)

POS advertising remains prominent in India and has been encountered for both smoked and SLT products [6]. It is done through oversized advertisement boards, placement of multiple advertisements on one board and placement of advertisement boards on shops not selling tobacco [8]. A 2‐year longitudinal trial undertaken to check the impact of tobacco advertisements among urban adolescents in Delhi and Chennai found that students exposed at more than 1.5 times (boys 1.7 times) more likely to progress towards tobacco use at endline versus those not exposed [15]. Substantial previous evidence of the impact of POS advertising was supported by data obtained from tobacco users in Mumbai, who confirmed that other forms of tobacco advertising were virtually non‐existent [6]. Noncompliance with Section 5 of COTPA regarding POS advertisement was reported in studies conducted in Karnataka state, where product showcases, stickers and posters were prominently used for promotion of tobacco products, more commonly in petty shops and urban areas [7]. This is a cause for concern, as suggestive evidence exists of a causal relationship between tobacco marketing and adolescent tobacco use [15].

FCTC Article 13 advocates a comprehensive advertising ban within 5 years of entry into the treaty. Currently, only 19 countries across the world (representing 6% of the global population) enforce a comprehensive ban on tobacco advertising, promotion and sponsorship (TAPS), with 101 countries (India) imposing partial bans and 74 countries having no ban at all [9]. While the COTPA Section 5 prohibits direct and indirect advertising (billboards, media) and sponsorship, exceptions still permit advertising at POS and on‐pack/in‐pack under specific regulations [6–8]. Despite regulations, high compliance issues exist at retail points, where advertising still occurs.

Tobacco packs are important means of advertising for the industry as they employ attractive logos, brand names, colours, etc. In a feasibility study for plain packaging of tobacco products conducted in Delhi, participants believed plain packaging would reduce attractiveness, appeal and promotional value of the pack, reduce experimentation and initiation of use among youth, and would motivate users to quit [8]. While India has implemented stringent health warnings covering 85% of the principal display area on both sides of tobacco product packages, it has not yet adopted the ‘plain packaging‘ standard that removes all brand imagery, logos and specific colours. Implementing plain tobacco packaging in India involves several ‘pitfalls‘ such as sale of loose cigarettes or individual sticks (thereby reducing visibility of warnings among consumers), legal challenges from the tobacco industry, diverse and fragmented tobacco market with a multitude of local products from unorganised cottage industries making it difficult to ensure regulatory compliance, resource constraints in enforcement agencies and low priority accorded to tobacco control.

At the endline, tobacco control shows moderate progress, but significant gaps remain due to weak enforcement and continued indirect promotion. These practices sustain product visibility and influence consumer behaviour. Strengthening enforcement, restricting POS displays, regulating the advertisement size and placement, and improving vendor awareness are essential to ensure better compliance and reduce tobacco use effectively.

### 3.4. Pictorial Warnings on Tobacco Products (Covered Under FCTC Article 11, COTPA Section 7)

Under the NTCP, a prominent measure taken to reduce tobacco consumption was the annual change in pictorial warnings on packs, but its impact on tobacco consumers is less studied [16, 17]. In an encouraging finding, a majority of patients attending a dental college in Bangalore city reported noticing the pictorial warnings and believed that they created awareness about health hazards and assisted in reducing or quitting the habit [17]. Awareness about the warnings was also significantly high among tobacco consumers in coastal Karnataka [18]. College students in Udupi district of Karnataka state recalled the warnings on packs and rated them as impactful in preventing initiation and reducing and stopping tobacco use. In addition, they opined that warnings on cigarette sticks may help complement warnings on cigarette packs and deter initiation [19]. An investigation carried out in a field practice area in coastal Andhra Pradesh, India, found more than 50% participants reporting that they never came across pictorial warnings, while 71.2% believed that the size and position of pictorial warnings were not sufficient to understand the harmful effects of tobacco [20]. In a study among tobacco users of different socioeconomic strata attending a dental college in Andhra Pradesh, a majority of participants stated that pictorial warnings need to carry stronger messages to make people quit tobacco and that graphic warnings should cover 100% of the pack to be more effective [21]. While 72.5% tobacco consumers of Bellary city, Karnataka, had seen the pictorial warnings, only 14.5% had attempted to quit [22]. Similarly, while a majority of current smokers visiting a Rural Health Center in Trichy, Tamil Nadu, reported noticing the pictorial warnings, a significant proportion among them were not motivated to quit. Since many purchased cigarettes in singles rather than whole pack, they could have missed viewing the pictorial warnings [16]. Similarly, high dependence tobacco users in Kanpur reported not being influenced by statutory warnings or anti‐tobacco advertisements [10]. Population‐level policies like price rise and pictorial warnings often fail for this group because severe nicotine addiction makes use a compulsive need. Intensive targeted measures such as clinical and pharmacological interventions, psychological support, access to peer support networks, etc. are necessary in this group. While pictorial warnings have heightened awareness about health hazards, they can be buttressed with warnings on cigarette sticks, as loose cigarette sales are common in India [19].

The evaluation of effectiveness of COTPA and NTCP showed varying scenarios in three Indian states. In Kerala, multiple stakeholders—government departments, regional cancer centres, media and civil society organisations—engaged in ‘collective action’ based on underlying trust, shared vision and effective resource pooling, leading to a widely accepted movement of tobacco control. In contrast, West Bengal and Arunachal Pradesh demonstrated high social acceptance and normalisation of tobacco use, sparse civil society engagement, non‐existent citizen participation, low awareness of the law and harms of tobacco use, and strong presence of the tobacco industry, leading to continued tobacco use [23].

In India, the informal nature of the beedi industry undermines national tobacco control policies because the deeply fragmented industry is woven into the rural economy. Challenges faced in regulatory enforcement include (a) Decentralised production distributed across millions of rural homes, making it difficult to monitor or enforce manufacturing regulations or health protections; (b) exploitative networks of middlemen or contractors who supply raw materials directly to rollers, thereby allowing evasion of statutory labour laws; (c) unbranded, loose distribution without warnings and labelling, bypassing COTPA guidelines; (d) persistent affordability due to fiscal policies which make beedis the cheapest smoked tobacco option in India, undermining efforts aimed at reducing consumption among youth and low‐income populations and (e) Political resistance to regulatory attempts as it is a critical source of employment for rural women.

Limitations of this study were that the narrative design may have caused selection bias that led to the potential omission of unpublished studies.

## 4. Conclusions

The tobacco epidemic kills over 8 million people annually and costs 5.7% of global health expenditure and 1.8% of global GDP, almost 40% of which is borne by the low and middle‐income countries (LMICs) [24]. While there has been unquestionable progress in tobacco control, implementation gaps and industry‐attributable challenges require multisectoral interventions for achieving full compliance with COTPA 2003 and the WHO FCTC [1].

Twenty‐five key departments of the GoI are involved in tobacco control. However, partnership with enforcement agencies, civil society organisations and the community at all levels is vital for strengthening enforcement of tobacco control laws [1, 23]. While implementation priority has been given to the WHO FCTC measures, Article 2.1 encourages implementation of measures beyond the treaty to provide ultimate protection of health [1, 24, 25].

In conclusion, four key tobacco‐control policies in India—price rise, smoke‐free legislation, bans on advertising and promotion, and use of PHWs—have collectively contributed to a measurable reduction in tobacco use. Among these,

1. The price rise through taxation remains the most effective intervention, especially in deterring initiation and encouraging cessation among price‐sensitive populations.
2. Smoke‐free laws have played a crucial role in reducing exposure to SHS both in homes and at workplaces.
3. Comprehensive bans on advertising and promotion have limited the reach of the tobacco industry, while indirect and surrogate marketing continue to pose challenges.
4. PHWs have improved awareness of tobacco‐related harms among youth and in the community, and their potential could be further enhanced through periodic updating and stronger compliance.

Despite these achievements, significant gaps persist in policy implementation, enforcement, and monitoring, limiting the full potential of these interventions. Regional disparities, industry interference, and the increasing availability of alternative tobacco products underscore the need for a more integrated approach—combining stronger enforcement, regular policy evaluation, public education and targeted interventions for vulnerable groups—to accelerate progress in tobacco control. Strengthening these measures within the public health framework will be critical in bridging the divide in India’s tobacco epidemic to reduce tobacco use and achieve long‐term gains. Complementing the MPOWER ‘Best Buys’ with Tobacco Endgame Strategies may provide critical breakthroughs in achieving a ‘Tobacco‐Free India’.

## Author Contributions

**Rekha P. Shenoy:** conceptualisation, data curation, formal analysis, funding acquisition, methodology, writing – original draft, writing – review and editing. **T. A. Abdul Salam:** conceptualisation, data curation, writing – original draft, writing – review andediting. **M. Alfiya:** data curation, methodology, writing – original draft, writing – review and editing. **Amanna Supriya:** data curation, writing – original draft.

## Funding

This study was self‐funded by the authors. The Article Processing Charge (APC) for publication was supported by Yenepoya (Deemed to be University).

## Disclosure

All authors have read and approved the final version of the manuscript. Rekha P. Shenoy had full access to all of the data in this study and takes complete responsibility for the integrity of the data and the accuracy of the data analysis.

## Ethics Statement

This narrative review is based on published articles available in the public domain and is a secondary data interpretation. Hence, ethical approval was not required.

## Conflicts of Interest

The authors declare no conflicts of interest.

## Data Availability

This narrative review is based on published articles available in the public domain and is a secondary data interpretation. The data supporting the findings are available and can be freely accessed.
